# Supplementary material for: Mechanistic and Structural Insights on Difluoromethyl-1,3,4-oxadiazole Inhibitors of HDAC6
Source: Int J Mol Sci. 2024 May 28;25(11):5885. doi: 10.3390/ijms25115885 (PMC11172862; doi:10.3390/ijms25115885)
Supplement: Supplementary file 1 [file ijms-25-05885-s001.zip › ijms-3004896-supplementary.pdf]

Supporting information

Table of Contents

KINETIC CHARACTERIZATION OF ITF7209 2
Selective HDAC6 inhibitor 4
CRYSTALLOGRAPHIC PARAMETERS 5
Experimental procedures 6
- **CHEMISTRY** 6
- **ENZYMATIC MEASUREMENTS** 12
  - **NMR experiment** 12
  - **HDAC activity assays** 13
  - **LC-MS experiments** 14
- **PROTEIN PRODUCTION AND X-RAY CRYSTALLOGRAPHY** 14
  - **Protein production and purification** 14
  - **Crystallization and Data Collection** 15
  - **Structure Determination** 15

REFERENCES 16

KINETIC CHARACTERIZATION OF ITF7209

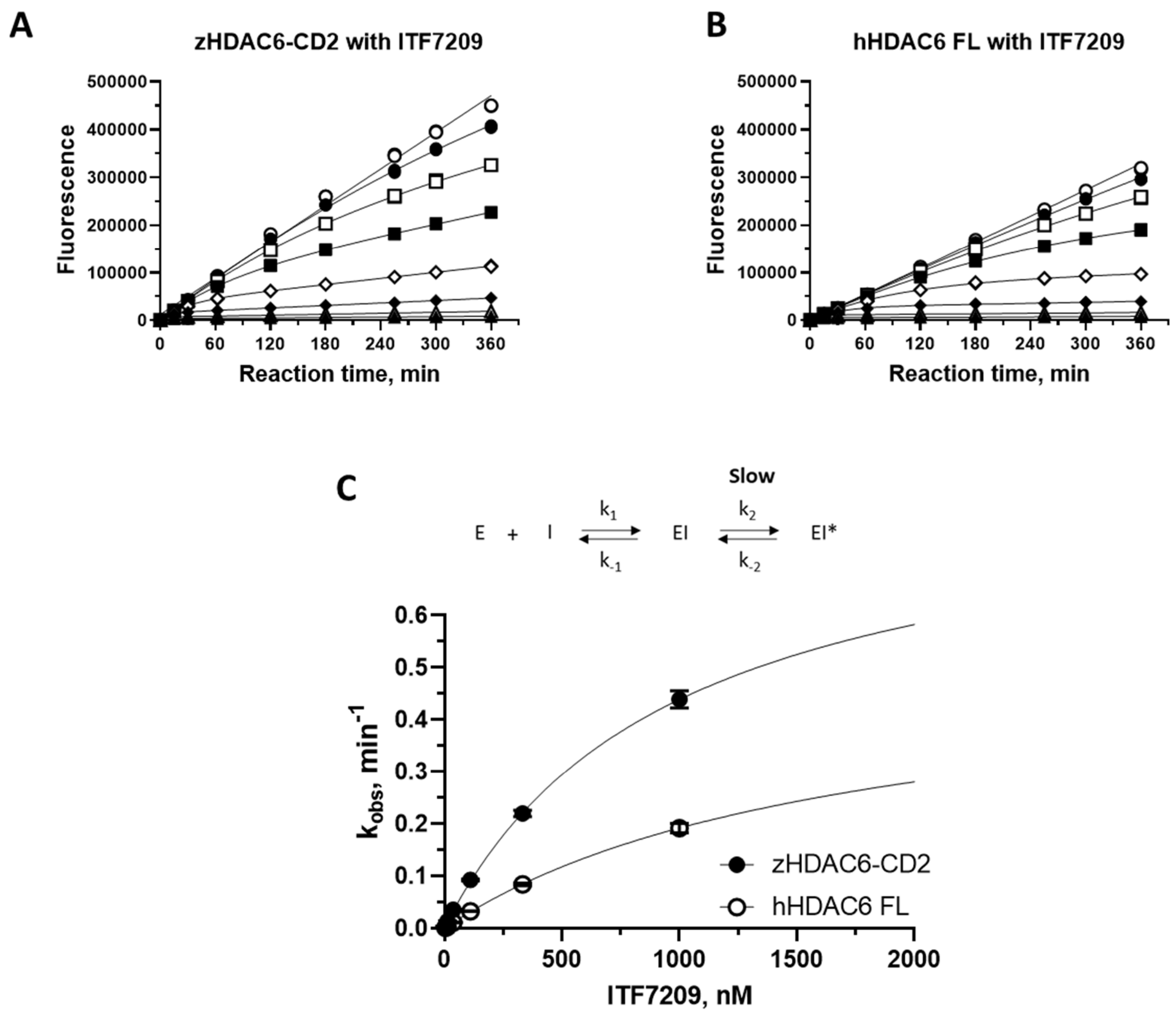

Figure S1. Kinetic evaluation of ITF7209 with zHDAC6-CD2 and human HDAC6 full-length.
The progress curves for zHDAC6-CD2 (A) and human HDAC6 full-length (B) in the presence of various concentrations of ITF7209 were carried out in assay buffer and with 3 μM Fluor-de-Lys Green. Concentration of ITF7209: 0 (○), 1.37 (●), 4.11 (□), 12.34 (■), 37.04 (◇), 111.11 (◆), 333.33 (△) and 1000 (▲) nM. The data were fitted to Eq. 1 to determine the k_{obs} values. (C) Replot of the k_{obs} values (with their associated error) for inhibition of HDAC6 as function of ITF7209 concentration yields a hyperbolic relationship. The experiments in panels A and B were carried out in duplicates and shown as scatter charts. In panel C, error bars represent the standard deviation (SD) of the mean values derived from Panels A and B. All the calculated kinetic values by fitting data to Eq. 2 are summarized in Table S1.

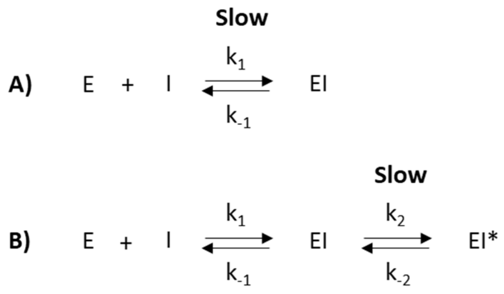

Figure S2. Slow binding inhibition mechanisms.
A) The one-step process resulting from an initial slow binding. **B)** Two-step process or induced-fit model. First, an EI complex is formed in rapid equilibrium with free enzyme and inhibitor. Then, such initial EI complex is slowly converted into a tighter EI* complex.

Table S1. Kinetic parameters of the inhibition of HDAC6 forms by ITF7209.

HDAC6 forms	K_i (µM)	k_2 (min^{-1})	k_{-2} (min^{-1})	K_i^* (nM)
**human HDAC6**	1.71 ± 0.05	0.52 ± 0.01	~ 10^{-4}	7.20 ± 0.06
**zHDAC6-CD2**	0.98 ± 0.04	0.86 ± 0.02	~ 10^{-3}	6.70 ± 0.04

Rate and equilibrium constants derived from the experiments shown in Figure S1. K_i is the dissociation constant of the initial EI complex. k_2 and k_{-2} are the rate constants describing the formation of the tight EI* complex. k_{-2} is an estimate value since it is very small and difficult to distinguish from zero. K_i^* is the dissociation constant of the tight EI complex.

Selective HDAC6 inhibitor

A - Motlová et al.

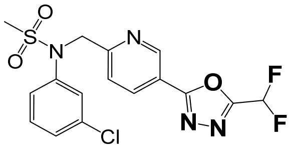

8

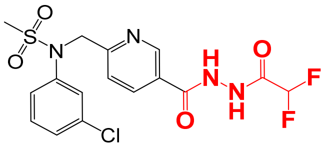

6

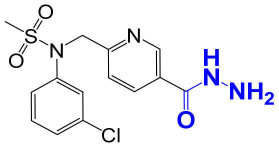

4

B - König et al.

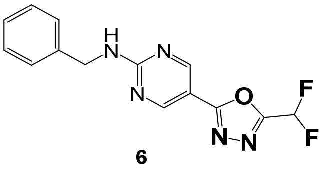

6

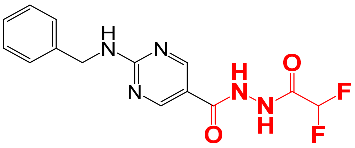

13

C - Ripa et al.

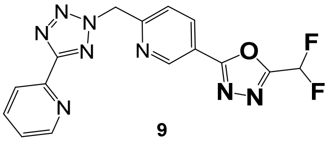

9

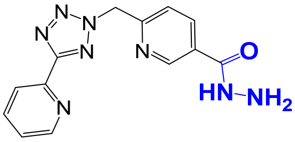

23

D - Vergani et al.

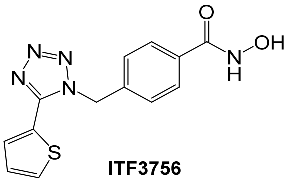

ITF3756

Figure S3. Structures of various HDAC6-selective inhibitors.
(**A**) Compounds 8, 6 and 4 are reported by Barinka and co-workers (1). (**B**) Compounds 6 and 13 are described by Konig et al. (2). (**C**) Compounds 9 and 23 are described by Ripa et al. (3)**.** (**D**) ITF3756 from (4)

CRYSTALLOGRAPHIC PARAMETERS

Table S2. Parameters for crystallographic data collection and refinement.

**zHDAC6-CD2-ITF7209 complex**	
Crystallization condition	0.1% (w/v) n-Octyl-β-D-glucoside, 0.1 M Sodium citrate tribasic dihydrate, pH 5.5, 21% (w/v) PEG 3350
**Data collection**	
Beamline	PROXIMA1 (SOLEIL)
Wavelength (Å)	0.979
Space group	$P2_12_12_1$
Cell dimensions: a, b, c (Å), α, β, γ (°)	74.7, 91.6, 96.1, 90.0, 90.0, 90.0
Resolution (Å)	48.06-1.86 (1.91-1.86)
R_{merge}	0.18 (1.44)
Mean I/σI	8.7 (1.8)
$CC_{1/2}$ (%)	99.7 (79.2)
Completeness (%)	99.9 (99.3)
Redundancy	12.9 (11.3)
Wilson *B* factor (Å^2)	27.1
**Refinement**	
Resolution range	48.06-1.86 (1.91-1.86)
No. of reflections	53202 (4074)
Completeness (%)	99.9 (99.3)
No. of non-H atoms	6076
R_{work}/R_{free} (R_{free} = 5% of reflections)	0.18/0.22 (0.30/0.35)
Mean *B*-value, overall (Å^2)	28.7
RMSD from ideal values - bond length (Å)/bond angle (°)	0.008/1.6
Correlation coefficient between F_o and F_c all/free	0.97/0.95
Ramachandran plot - favored/allowed/outliers (%)	96/4/0

Values in parenthesis are for the highest resolution shell.

Experimental procedures

CHEMISTRY

Synthesis of N-{4-[1-({4-[5-(difluoromethyl)-1,3,4-oxadiazol-2-yl] phenyl} methyl)-1H-1,2,3-triazol-4-yl] phenyl}-4,5-dihydro-1H-imidazol-2-amine (ITF5924).

Step A

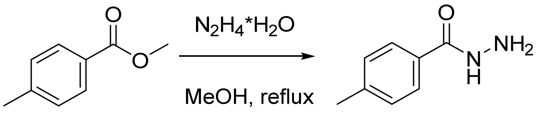

Methyl 4-methylbenzoate (70 g, 466 mmol, 1 equiv.) was dissolved in 350 mL MeOH, then hydrazine monohydrate was added (2.5 equiv.) under stirring. The mixture was refluxed overnight. Full conversion of methyl ester to hydrazide was observed by LCMS (and TLC). The reaction mixture was concentrated and dried under vacuum. The white solid obtained (70 g, 99% yield) was used for the subsequent step without further purification.

Step B

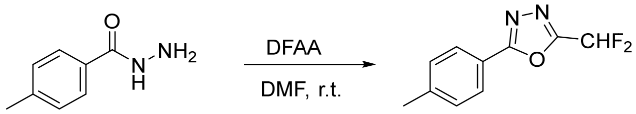

The hydrazide obtained in step A (50 g, 333 mmol, 1 equiv.) was dissolved in dry DMF (300 mL) under argon. Difluoroacetic anhydride (3 equiv.) was slowly added through a dropping funnel, keeping temperature below 10°C (ice/NaCl bath). After addition was complete, the temperature was let reach r.t.. The flask was sealed, and the reaction mixture was stirred at r.t. overnight. Conversion was observed by LCMS.
The reaction mixture was carefully poured into sat. aq. $NaHCO_3$ to quench the DFAA excess. The product precipitated as a solid and could be collected by filtration. After removing residual solvents, the crude product was dissolved in MTBE, washed 3 times with 300 mL of $NaHCO_3$ and brine. Organic phase was dried over Na_2SO_4, filtered, and evaporated to give a crude product (43 g, 205 mmol, 61% yield), which was used in the next step without further purification.

Step C

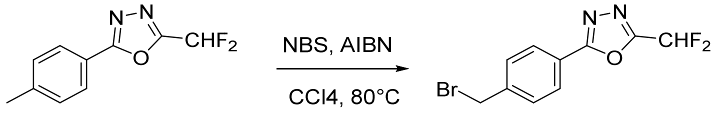

2-(difluoromethyl)-5-(4-methylphenyl)-1,3,4-oxadiazole (43 g, 205 mmol, 1 equiv.) was dissolved in 800 ml degassed carbon tetrachloride. *N*-Bromosuccinimide (NBS, 1.05 equiv.) and azobisisobutyronitrile (AIBN, 0.01 equiv.) were added to the reaction mixture, which was stirred at 75°C over 4h.
The mixture was diluted with DCM, washed with water (3x200 mL), $NaHCO_3$ (200 mL) and brine (2x200 mL). The organic phase was dried over Na_2SO_4, filtered, and evaporated to give a crude product (80 g). The crude residue was reprecipitated from DCM/hexane, affording pure product (36 g, 125 mmol, 61% yield).

Step D

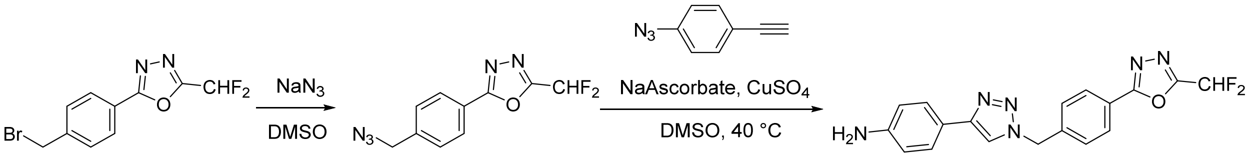

A solution of 2-[4-(bromomethyl)-phenyl]-5-(difluoromethyl)-1,3,4-oxadiazole (1 g, 3.46 mmol, 1 equiv.) and sodium azide (1 equiv.) in 10 mL DMSO was stirred at r.t. for 1 h. Full conversion to intermediate 2-(4-(azidomethyl)-phenyl)-5-(difluoromethyl)-1,3,4-oxadiazole was observed by HPLC.
4-Ethynylaniline (405 mg, 1 equiv.) was then added to the reaction mixture, followed by copper(II) sulfate pentahydrate (0.2 equiv., 0.5 M aqueous solution) and sodium L-ascorbate (0.4 equiv., 1 M aqueous solution). The reaction mixture was agitated at 40°C overnight. Full conversion of the starting material was detected by LC-MS. Water was added to the reaction mixture, and a precipitate formed. The precipitate was filtered and washed with water. Purification by flash chromatography (silica gel, Hex:EtOAc 0-100%) gave the desired product in good purity (973 mg, 2.61 mmol, 75% yield).

Step E

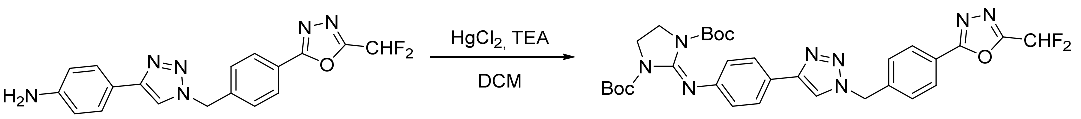

$HgCl_2$ (1.1 equiv.) was added to a solution of 4-(1-(4-(5-(difluoromethyl)-1,3,4-oxadiazol-2-yl)benzyl)-1H-1,2,3-triazol-4-yl)aniline (84 mg, 0.228 mmol, 1 equiv.), N,N'-di(*tert*butoxycarbonyl)imidazolidine-2-thione (1 equiv.) and triethylamine (1.3 equiv.) in 1 mL DCM at 0°C. The resulting mixture was stirred at 0°C for 1 h and at r.t. for 2 days. The reaction mixture was diluted with water and DCM, filtered, and extracted with DCM. The organic layer was washed with brine, dried over $MgSO_4$, filtered, and concentrated under reduced pressure to afford a yellow oil, which was used directly in the next step (145 mg, 0.228 mmol, 100% yield).

Step F

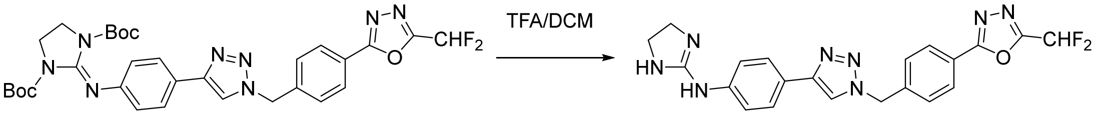

Di-*tert*-butyl 2-((4-(1-(4-(5-(difluoromethyl)-1,3,4-oxadiazol-2-yl)benzyl)-1H-1,2,3-triazol-4-yl)phenyl)imino)imidazolidine-1,3-dicarboxylate (145 mg, 0.228 mmol, 1 equiv.) was dissolved in 2 mL DCM and TFA (20 equiv.) was added. The reaction mixture was stirred at r.t. overnight. The mixture was diluted with DCM and washed with sat. aq. $NaHCO_3$ and brine. During washing with brine, precipitation occurred. The solid was filtered, washed with water and dried under vacuum to obtain the desired product (55 mg, 0.121 mmol, 53% yield). ^{1}H NMR (400 MHz, DMSO) δ 8.75 (s, 1H), 8.20 (d, 2H), 7.90 (d, 2H), 7.60 (s, 2H), 7.55 (t, 1H), 7.30 (d, 2H), 5.78 (s, 2H), 3.65 (s, 4H). LCMS (ESI+) calculated for $C_{21}H_{18}F_2N_8O$ $[M+H]^+$: 437.42; found: 437.12.

Synthesis of N'-(2,2-difluoroacetyl)-4-((4-(4-((4,5-dihydro-1H-imidazol-2-yl)amino)phenyl)-1H-1,2,3-triazol-1-yl)methyl)benzohydrazide (ITF6715).

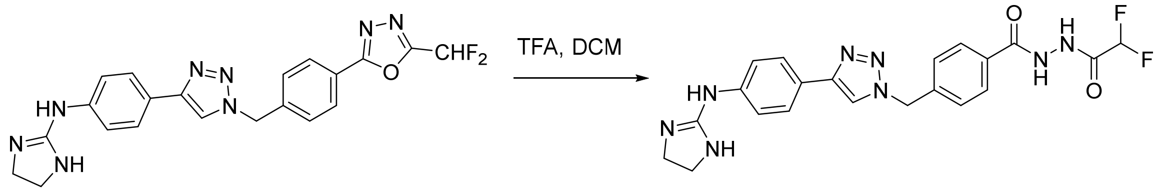

N-[4-[1-[[4-[5-(difluoromethyl)-1,3,4-oxadiazol-2-yl]phenyl]methyl]triazol-4-yl]phenyl]-4,5-dihydro-1H-imidazol-2-amine (50 mg, 0.115 mmol, 1 equiv.) was suspended in 1 mL of a 1:1 water/ACN mixture and TFA (40 equiv.) was added. The reaction mixture was stirred at r.t. overnight. Full conversion of the starting material was observed by LCMS.

The reaction mixture was purified by prep HPLC (ACN + 0.1% FA /H_2O + 0.1% FA) affording 20 mg of the desired product (0.043 mmol, 37% yield). ^{1}H NMR (400 MHz, DMSO) δ 10.25 (s, 4H), 8.60 (s, 1H), 8.30 (s, 1H, HCOOH), 7.80 (dd, 4H), 7.45 (d, 2H), 7.25 (d, 2H), 6.30 (t, 1H), 5,75 (s, 2H), 3.55 (s, 4H). LCMS (ESI+) calculated for $C_{21}H_{20}F_2N_8O_2$ $[M+H]^+$: 455.43; found: 455.23.

Synthesis of 4-((4-(4-((4,5-dihydro-1H-imidazol-2-yl)amino)phenyl)-1H-1,2,3-triazol-1-yl)methyl)benzohydrazide (ITF6712).

Step A

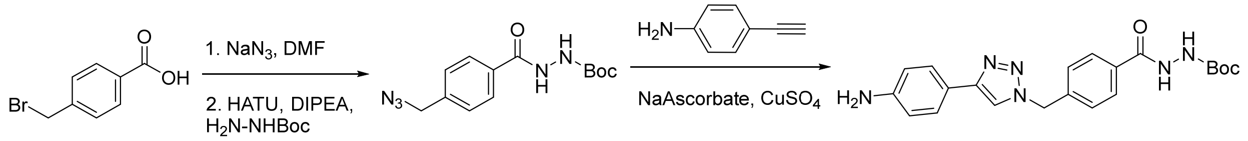

A solution of 4-(bromomethyl)benzoic acid (1 g, 4.65 mmol, 1 equiv.) and sodium azide (1 equiv.) in 20 mL DMF was stirred at r.t. for 1 h. HATU (1.1 equiv.), *N,N*-diisopropylethylamine (2 equiv.) and tert-butyl hydrazinecarboxylate (1 equiv.) were added; the reaction mixture was stirred at r.t. overnight. Conversion of the starting material was monitored by LCMS. Copper(II) sulfate pentahydrate (0.15 equiv., 0.5 M aqueous solution) and sodium L-ascorbate (0.3 equiv., 1 M aqueous solution) were added. The reaction mixture was stirred at r.t. overnight; full conversion to the desired product was observed by LCMS.

Upon dilution with water the product precipitated as a solid (1.76 g, 4.32 mmol, 92% yield) which was used directly in the next step.

Step B

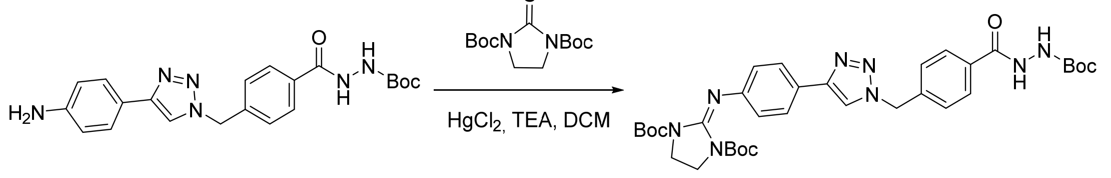

$HgCl_2$ (1.1 equiv.) was added to a solution of tert-butyl 2-(4-((4-(4-aminophenyl)-1H-1,2,3-triazol-1-yl)methyl)benzoyl)hydrazine-1-carboxylate (786 mg, 1.92 mmol, 1 equiv.), di*tert*-butyl 2-sulfanylideneimidazolidine-1,3-dicarboxylate (1.1 equiv.) and triethylamine (3 equiv.) in 10 mL DCM. The reaction mixture was stirred at r.t. over 48 h. Full conversion of the starting material was detected by LCMS.
The reaction mixture was diluted with DCM and filtered on a Celite pad. The filtrate was washed with brine (3x), dried over Na_2SO_4, filtered, and concentrated under reduced pressure to afford a yellow/orange solid (1.30 g, 1.92 mmol, 99% yield) which was used in the next step without any further purification.

Step C

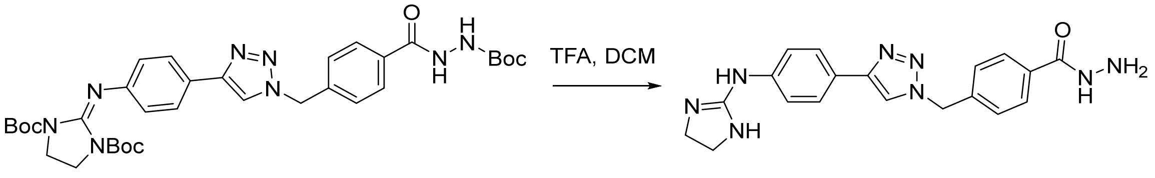

Di*tert*-butyl 2-[4-[1-[[4-[[(2-methylpropan-2-yl)oxycarbonylamino]carbamoyl]phenyl]methyl]triazol-4-yl]phenyl]iminoimidazolidine-1,3-dicarboxylate (650 mg, 0.96 mmol, 1 equiv.) was dissolved in 4 mL DCM and TFA (30 equiv.) was added. The reaction mixture was stirred at r.t. overnight. The full conversion of the starting material was monitored by LCMS.

The reaction mixture was neutralized with $NaHCO_3$; the organic solvent was removed under reduced pressure. The crude residue was purified by RP Prep-HPLC (H_2O/ACN: 95/5 to 80/20). The desired product was isolated as a white solid (140 mg, 0.37 mmol, 38% yield). ^{1}H NMR (400 MHz, DMSO) δ 9.75 (s, 1H), 8.75 (s, 1H), 8.30 (s, 2H), 7.80 (dd, 4H), 7.30 (dd, 4H), 5.70 (s, 2H), 4.50 (s, 2H), 3.70 (s, 4H). LCMS (ESI+) calculated for $C_{19}H_{20}N_8O$ $[M+H]^+$: 377.42; found: 377.23.

Synthesis of 2-[5-[(4-tert-butyltriazol-1-yl)methyl]thiophen-2-yl]-5-(difluoromethyl)-1,3,4-oxadiazole (ITF7209).

Step A

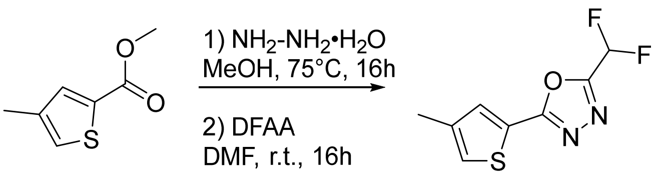

Methyl 4-methylthiophene-2-carboxylate (1 g, 6.4 mmol, 1 equiv.) was dissolved in 15 mL methanol and hydrazine hydrate (4 equiv.) was added. The resulting mixture was stirred at 75°C overnight. The starting material was fully converted to the intermediate hydrazide. The reaction mixture was concentrated under reduced pressure and the residual white solid was dried overnight.

The crude hydrazide was dissolved in 10 mL DMF under argon, and the resulting solution was cooled down to 0°C. Difluoroacetic anhydride (2 equiv.) was added dropwise; then, the mixture was allowed to reach r.t. and was stirred at r.t. overnight. Water was added to the reaction mixture, which was extracted with EtOAc (3x200 mL). The combined organic layer was washed with sat. aq. $NaHCO_3$ and brine, dried ($MgSO_4$), filtered and concentrated under reduced pressure. The residue was purified by flash chromatography (hexane/EtOAc, 95:5 to 7:3) affording the product as a colorless oil/solid (727 mg, 3.36 mmol, 52% yield).

Step B

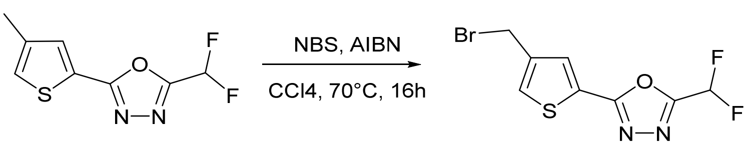

A mixture of 2-(difluoromethyl)-5-(4-methylthiophen-2-yl)-1,3,4-oxadiazole (581 mg, 2.69 mmol, 1 equiv.) and N-bromosuccinimide (1.05 equiv.) in 10 mL carbon tetrachloride was stirred under argon until complete dissolution. Then AIBN (0.03 equiv.) was added to the reaction mixture, which was stirred at 70°C overnight. The mixture was then allowed to reach r.t., diluted with DCM and washed successively with sat. aq. $NaHCO_3$, water and brine. The organic layer was separated, dried over $MgSO_4$, filtered, and concentrated under reduced pressure. The residue was purified by flash chromatography (silica gel, hexane/EtOAc, 9:1 to 8:2) affording the product as white solid (405 mg, 1.37 mmol, 51% yield).

Step C

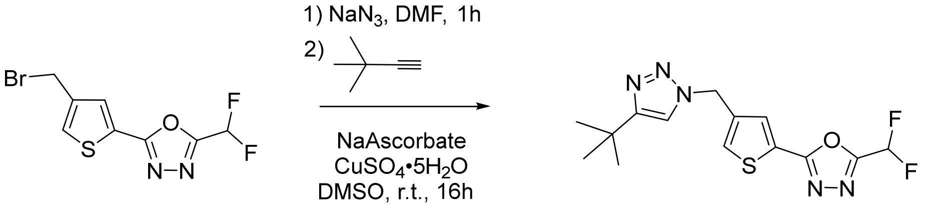

2-(4-(bromomethyl)thiophen-2-yl)-5-(difluoromethyl)-1,3,4-oxadiazole (70 mg, 0.24 mmol, 1 equiv.) and sodium azide (1.1 equiv.) were dissolved in DMSO and the reaction mixture was stirred at r.t. over 1 hour. 3,3-dimethylbut-1-yne (1 equiv.) was then added, followed by addition of sodium L-ascorbate (1 M, 0.5 equiv.) and copper(II) sulfate pentahydrate (0.5 M, 0.3 equiv.) aqueous solutions. The reaction mixture was stirred at r.t. for 2h. Water was added to the mixture and precipitation of the product occurred. The precipitate was collected by filtration and washed with water. The crude thus obtained was purified by prep-HPLC (water/ACN + 0.1% FA) affording the product as a white solid (18.62 mg, 0.05 mmol, 98.00% purity, 23% yield). ^{1}H NMR 400 MHz, DMSO-d6) δ 8.00 (s, 1H), 7.84 (d, J = 3.8 Hz, 1H), 7.52 (t, J = 51.3 Hz, 1H), 7.34 (d, J = 3.8 Hz, 1H), 5.88 (s, 2H), 1.27 (s, 9H); LCMS (ESI+) calculated for $C_{14}H_{15}F_2N_5OS$ $[M+H]^+$: 340.36, found: 340.15.

Synthesis of 5-[(4-tert-butyltriazol-1-yl)methyl]-N'-(2,2-difluoroacetyl)thiophene-2-carbohydrazide (ITF7738)

Step A

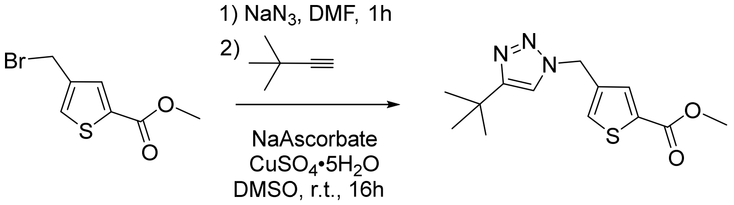

Methyl 5-(bromomethyl)thiophene-2-carboxylate (500 mg, 2.13 mmol, 1 equiv.) and sodium azide (1.1 equiv.) were dissolved in 5 mL DMSO and the reaction mixture was stirred at r.t. over 1 hour. 3,3-dimethylbut-1-yne (3 equiv.) was then added, followed by addition of sodium L-ascorbate (1 M, 0.2 equiv.) and copper(II) sulfate pentahydrate (0.5 M, 0.4 equiv.) aqueous solutions. The reaction mixture was stirred at r.t. overnight. Ammonium solution was added to the mixture, which was extracted with EtOAc (3x200 mL). The organic layer was separated, dried over $MgSO_4$, filtered, and concentrated under reduced pressure. The crude (473 mg, 1.69 mmol, 80% yield) was used in the next step without any further purification.

Step B

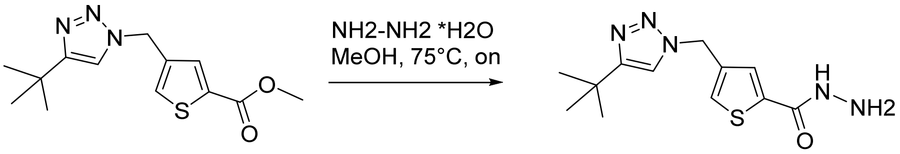

The crude methyl 5-[(4-tert-butyltriazol-1-yl)methyl]thiophene-2-carboxylate (473 mg, 1,69 mg, 1 equiv.) was dissolved in 10 mL methanol and hydrazine hydrate (4 equiv.) was added. The resulting mixture was stirred at 75°C overnight. The starting material was fully converted to the intermediate hydrazide. The reaction mixture was concentrated under reduced pressure and the residual white solid was dried overnight. The crude (383 mg, 1.30 mmol, % yield) was used in the next step without any further purification.

Step C

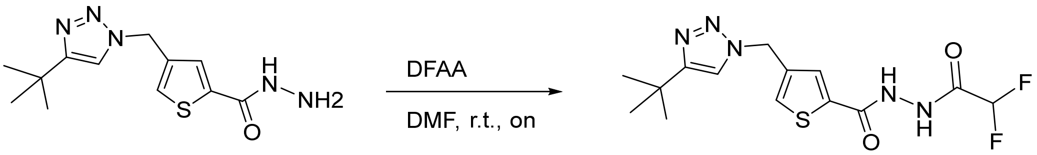

5-[(4-tert-butyltriazol-1-yl)methyl]thiophene-2-carbohydrazide (383 mg, 1.30 mmol, 1 equiv.) was dissolved in 7 mL DMF under argon, and the resulting solution was cooled down to 0°C. Difluoroacetic anhydride (1 equiv.) was added dropwise; then, the mixture was allowed to reach r.t. and was stirred at r.t. overnight. The mixture was diluted with $NaHCO_3$ solution, and then extracted with AcOEt (3x100 mL). The organic layer was separated, dried over $MgSO_4$, filtered, and concentrated under reduced pressure. The crude was purified by prep-HPLC (water/ACN + 0.1% FA) affording the product as a white solid (75.49 mg, 0.21 mmol, 98.56% purity, 16% yield). ^{1}H NMR (400 MHz, DMSO) δ 10.96 (br s, 1H), 10.69 (br s, 1H), 7.95 (s, 1H), 7.72 (d, J = 3.8 Hz, 1H), 7.22 (d, J = 3.8 Hz, 1H), 6.44 (t, J = 52.9 Hz, 1H), 5.79 (s, 2H), 1.27 (s, 9H); LCMS (ESI+) calculated for $C_{14}H_{17}F_2N_5O_2S$ $[M+H]^+$: 358.38; found: 358.25.

Synthesis of 2 5-[(4-tert-butyltriazol-1-yl)methyl]thiophene-2-carbohydrazide (ITF7739)

Step A

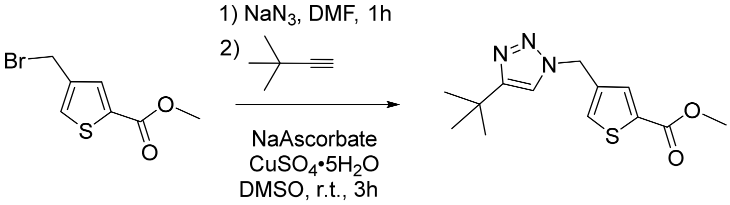

Methyl 5-(bromomethyl)thiophene-2-carboxylate (464 mg, 1,97 mmol, 1 equiv.) and sodium azide (1.1 equiv.) were dissolved in 5 mL DMSO and the reaction mixture was stirred at r.t. over 1 hour. 3,3-dimethylbut-1-yne (3 equiv.) was then added, followed by addition of sodium L-ascorbate (1 M, 0.4 equiv.) and copper(II) sulfate pentahydrate (0.5 M, 0.2 equiv.) aqueous solutions. The reaction mixture was stirred at r.t. overnight. Ammonium solution was added to the mixture, which was extracted with EtOAc (3x200 mL). The organic layer was separated, dried over $MgSO_4$, filtered, and concentrated under reduced pressure. The crude (551 mg, 1.97 mmol, 100% yield) was used in the next step without any further purification.

Step B

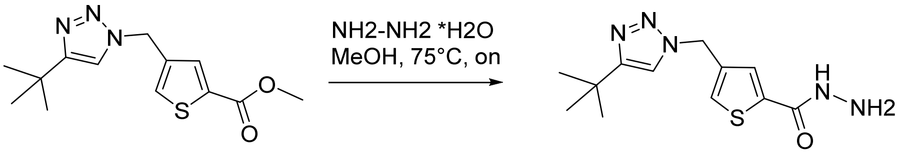

The crude methyl 5-[(4-tert-butyltriazol-1-yl)methyl]thiophene-2-carboxylate (551 mg, 1,97 mg, 1 equiv.) was dissolved in 10 mL methanol and hydrazine hydrate (10 equiv.) was added. The resulting mixture was stirred at 75°C overnight. The starting material was fully converted to the intermediate hydrazide. The reaction mixture was concentrated under reduced pressure, and the crude brown product was extracted with EtOAc (3x 150 mL). The organic layer was washed with brine, dried over $MgSO_4$, filtered, and concentrated under reduced pressure. The crude was purified by prep-HPLC (water/ACN 0.1% FA) to afford the product as white solid (118 mg, 0.42 mmol, 20% yield). ^1H NMR (300 MHz, DMSO-d_6) δ 9.77 (s, 1H), 7.92 (s, 1H), 7.57 (d, J = 3.8 Hz, 1H), 7.14 (d, J = 3.8 Hz, 1H), 5.74 (s, 2H), 4.50 (s, 2H), 1.27 (s, 9H); LCMS (ESI+) calculated for $C_{12}H_{17}N_5OS$ $[M+H]^+$: 280.36; found: 280.16.

ENZYMATIC MEASUREMENTS

NMR experiment

The zHDAC6-CD2 storage buffer was replaced with a deuterated buffer (containing 25 mM TRIS-d_{11}, and 0.5 mM TCEP-d_{16} in D_2O; pH adjusted to 8.0 with DCl) by centrifugal ultrafiltration using Vivaspin 500 concentrators (Sartorius, Göttingen, Germany) equipped with 10 kDa MWCO membranes. An aliquot of zHDAC6-CD2 solution (125 µL; 1.15 mg/mL; 28.5 µM) was repeatedly washed with ten 125-µL aliquots of deuterated buffer. The final zHDAC6-CD2 solution (140 µL) had a concentration of 0.925 mg/mL (23 µM; 90% recovery).

Protein concentrations were determined with a NanoDrop spectrophotometer (Thermo Fischer Scientific, Waltham, Massachusetts, USA) by measuring the absorbance at 280 nm (A_{280}) on 2-µL aliquots of undiluted protein solution. Concentrations were calculated using a theoretical extinction coefficient (ε_{280}) of 38390 $L \cdot mol^{-1} \cdot cm^{-1}$ and a molecular mass of 40234.53 Da.

ITF5924 was first dissolved in DMSO-d_6 at a concentration of 100 mM (stock solution). Analytical samples were prepared by diluting 11 µL of ITF5924 stock solution and 120 µL of zCD2-HDAC6 solution with 419 µL of deuterated buffer, reaching a final concentration of 2 mM and 5 µM for ITF5924 and zCD2-HDAC6, respectively. The total sample volume in the 5-mm NMR tube was 550 µL.

Nuclear magnetic resonance (NMR) spectra were acquired on a Bruker Avance III 600 MHz spectrometer (Bruker, Germany) equipped with a TXI probe. The Bruker library sequence noesygppr1d was used for ^1H NMR spectra acquisition, allowing an almost complete suppression of the residual HDO signal. Spectra were acquired at 278 K with 64 scans, a spectral with of 15 ppm, an acquisition time of 3.6 s and a relaxation delay of 5 s. Spectra were collected at 30 min time intervals over 72 h. After Fourier transformation, phase adjustment, and baseline correction, the chemical shift axis was calibrated using the CHD_2 signal at 2.50 ppm.

Reference spectra were acquired on samples containing ITF5924, ITF6712, ITF6715, or the enzyme alone under the same experimental conditions. The chemical stability of the inhibitors in the reaction buffer (i.e., without the enzyme) was verified over the same time interval (72 h).

Quantification of ITF5924, ITF6712, and ITF6715 was achieved by integrating three non-overlapping signals at 8.24, 8.20, and 8.18 ppm, respectively (Figure S4), which correspond to the CH group of the triazole moiety in each of the three molecules.

The software TopSpin (version 3.6.4, Bruker, Germany) was used for spectral acquisition, spectral processing, and signal integration. After processing, representative spectra were exported as text files and imported into GraphPad Prism (version 10.2.2, La Jolla, CA, USA) to generate Figure S4. GraphPad Prism was also used to generate Figure 3 using integrated signal areas at selected time points.

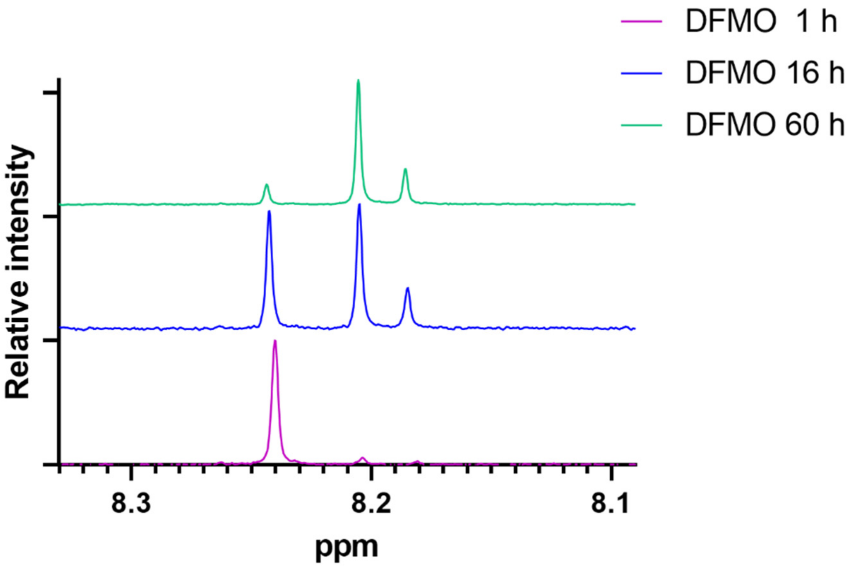

Figure S4. ^{1}H NMR spectra of the reaction mixture at 1 h (violet), 16 h (blue), and 60 h (green). Signals at approximately 8.24, 8.20, and 8.18 ppm were assigned to the triazole CH group of ITF5924, ITF6712, and ITF6715 respectively.

HDAC activity assays

Optimized fluorometric enzymatic assays were used as previously reported (5, 6). Briefly, the time-course of the reaction was monitored at 25 °C in HDAC assay buffer [25 mM Tris/HCl, pH 8.0, 130 mM NaCl, 0.05% Tween-20, 10% Glycerol, 1 mg/mL bovine serum albumin (BSA), 0.5 mM tris(2-carboxyethyl)phosphine (TCEP) and 1% DMSO]. Human HDAC1 (Catalog 50051, *BPS Bioscience*, San Diego, CA, USA), human HDAC6 (Catalog 50006, *BPS Bioscience*, San Diego, CA, USA) and the second catalytic domain of zebrafish HDAC6 (zHDAC6-CD2) were used at catalytic concentrations of 710 pM, 62.5-125 pM and 0.12 nM, respectively. The Fluor de Lys Green substrate (*Enzo Life Sciences*, Farmingdale, New York, USA) was included at a concentration close to the K_m value, 3 µM for HDAC6 forms and 25 µM for HDAC1. For fast binding inhibitors, the initial velocity (v_o) was calculated as (ΔF/min) from the slope of the straight line fitting the data points. In the case of slow binding inhibitors, the data from progress curves were fitted to Eq. 1 to obtain the initial velocity (v_i), the steady-state velocity at long reaction times (v_s), and the apparent first-order rate constant (k_{obs}). In Eq 1, P is the product concentration, expressed as fluorescence intensity and t is the reaction time (in minutes) (7). All progress curves are generated by using GraphPad Prism software (version 10.2.2, La Jolla, CA, USA). For IC_{50} determination, v_o for fast-on inhibitors or v_s for slow-on compounds were used to calculate the inhibition, as percentage with respect to the value

obtained in the absence of inhibitor. Such inhibition data were fitted in the GraphPad Prism software (version 10.2.2, La Jolla, CA, USA) using nonlinear regression analysis.

Eq. 1 $P = v_s \cdot t + (v_i - v_s)[1 - exp(-k_{obs} \cdot t)] / k_{obs}$

In Eq. 1, P is the product concentration, expressed as fluorescence intensity, and t is the reaction time (in minutes) (7). For the determination of IC_{50} values, v_o for fast-on inhibitors or v_s for slow-on compounds were used to calculate the inhibition, as percentage with respect to the value obtained in the absence of inhibitor. Such inhibition data were fitted in GraphPad Prism software (version 10.2.2, La Jolla, CA, USA) using nonlinear regression analysis.

For the slow onset of inhibition of HDAC6 forms in the presence of ITF7209, the dependence of the k_{obs} values on inhibitor concentration was used to determine the mechanism of inhibition. "One-step" or a "two-step" (or "induced-fit) processes are the most common mechanisms of slow binding inhibition (Figure S2) and the resulting "k_{obs} plots" should be linear for mechanism A and hyperbolic in the case of mechanism B (7). k_{obs} were replotted as a function of ITF7209 concentration and fitted to equation 2, assuming the validity of mechanism B, where K_i is the dissociation constant of the initial EI complex, whereas k_2 and k_{-2} are the rate constants describing the formation of the tight EI* complex. IC_{50} reflects the dissociation constant of the EI* complex (Ki^*).

Eq. 2 $k_{obs} = k_{-2} + \{k_2 \cdot [I]/(K_i + [I])\}$

LC-MS experiments

zHDAC6-CD2 (5 µM) was incubated at 25 °C with 5 µM ITF5924 or ITF7209 in HDAC assay buffer for 1 minute to allow complex formation. After that time, ITF3756 (4) (100 µM, final concentration) was added as competitive inhibitor. A parallel control sample lacking ITF3756 (0.5 % DMSO) was also prepared. At different times after competitor addition (0.5, 10, 20, 30, 60, 120, 240 and 480 min), aliquots (40 µL) were transferred to test tubes containing acetonitrile (200 µL) to quench the reaction. Samples were kept frozen at -80 °C until the LC-HRMS analysis. The experiment was performed in triplicate. Quantitation of DFMO parent compound (ITF5924, ITF7209), difluoro-acetylhydrazide (ITF6715, ITF7738) and hydrazide (ITF6712, ITF7739) derivatives in the samples was performed with respect to calibration curves obtained with varying concentrations of each compound. Raw data were then imported into GraphPad Prism (version 10.2.2, La Jolla, CA, USA) to generate Figure 4.

The LC-HRMS analysis was carried out using a Vanquish Flex UHPLC (Thermo Fisher Scientific, Waltham, Massachusetts, USA) and a high-resolution mass spectrometer Orbitrap QExactive Focus (Thermo Fisher Scientific, Waltham, Massachusetts, USA), equipped with a Heated Electrospray Ionization, operated in positive mode. A Full Scan analysis was set in the m/z range 50-500 amu. A XSelect HSS T3 50x2.1 mm, 2.5 µm chromatographic column (Waters, Milford, Massachusetts, USA) was used. Mobile phase A consisted of 0.1% formic acid in water and mobile phase B in 0.1% formic acid in acetonitrile. The flow rate was set to 0.5 mL/min, with a gradient program from 3 to 20% B in 3 minutes. The software Chromeleon CDS (version 7.3.2, Thermo Fisher Scientific, Waltham, Massachusetts, USA) was used for acquisition, processing, and signal integration.

PROTEIN PRODUCTION AND X-RAY CRYSTALLOGRAPHY

Protein production and purification

The second catalytic domain (CD2) of zHDAC6 was produced recombinantly from a nucleotide sequence encoding residues 440-798 of zHDAC6 and an N-terminal TEV-cleavable 8xHis-MBP tag. The sequence was codon-optimized for expression in *E. coli*, synthesized, and cloned into a

kanamycin-resistance expression vector (GenScript, Ryswick, Netherlands). The protein was expressed and purified as previously described (5). Briefly, the expressions were carried out in 2xYT medium supplemented with 50 mg/mL kanamycin and 1 mM $ZnSO_4$. The bacteria were cultured at 37 °C until OD_{600} = 1, when the temperature was reduced to 16 °C and protein expression was induced with 75 µM isopropyl β-d-1-thiogalactopyranoside (IPTG), after which the cultures were grown for additional 20 hours. The bacterial cells were harvested and resuspended in buffer A (50 mM Tris pH 8.0, 500 mM NaCl, 10% (v/v) glycerol, 2 mM TCEP, 50 mM L-arginine, 50 mM L-glutamate) supplemented with 10 µg/mL DNase I and protease inhibitor cocktail and lysed by sonication. The lysate was centrifuged, and recombinant protein was purified from the soluble fraction using Ni-charged Chelating Sepharose Fast Flow (Cytiva, Uppsala, Sweden) according to the manufacturer's instructions, with buffer A supplemented with 30 mM imidazole as binding buffer and buffer A supplemented with 300 mM imidazole as elution buffer. The eluted protein was subjected to size-exclusion chromatography (SEC) on a HiLoad 26/600 Superdex 200 column (GE Healthcare, Chicago, Illinois, USA), with buffer A as the running buffer. Fractions containing monomeric, non-aggregated protein were treated with TEV protease (30:1 mass ratio), and the cleaved 8xHis-MBP tag and TEV protease were separated from zHDAC6-CD2 on Dextrin Sepharose (GE Healthcare, Chicago, Illinois, USA) and Ni-charged Chelating Sepharose Fast Flow (Cytiva, Uppsala, Sweden) using buffer A. The protein was subjected to final SEC on a HiLoad 26/600 Superdex 200 column with buffer B (50 mM HEPES pH 7.5, 100 mM KCl, 5% (v/v) glycerol, 2 mM TCEP) as the running buffer. The fractions containing the protein were pooled, concentrated to 13 mg/ml, frozen in liquid nitrogen, and stored at -80 °C.

Crystallization and Data Collection

zHDAC6-CD2 at 300 µM was mixed with an excess of compound ITF7209 (final concentration: 2 mM). The crystallization trays were set up immediately after mixing. Screening of crystallization conditions was performed using the sitting drop vapor diffusion technique, 96-well 3-drop conical crystallization plates (Swissci, Neuheim, Switzerland), Crystal Gryphon Nanodispender (Art Robbins Instruments, Sunnyvale, CA, USA), and commercially available crystallization kits (Molecular Dimensions, Hampton Research, Anatrace). The prepared complex was mixed with the reservoir solution at a 1:1 ratio, and the resulting drop was equilibrated against 30 µL of the reservoir solution at 4 °C. Crystals of zHDAC6-CD2-ITF7209 complex were obtained in 0.1% (w/v) n-octyl-β-D-glucoside, 0.1 M Sodium citrate tribasic dihydrate, pH 5.5, and 21% (w/v) PEG 3350. The crystals were cryoprotected with 20% (v/v) Glycerol and flash-frozen in liquid nitrogen. Data collection was performed at PROXIMA 1 beamline (SOLEIL synchrotron, France) at 100 K. Data were indexed and integrated using XDS and scaled in XSCALE (8). The best crystal diffracted to 1.9 Å, belonged to space group $P2_12_12_1$, and contained two protein molecules per asymmetric unit.

Structure Determination

The crystal structure of the zHDAC6-CD2-ITF7209 complex was determined by molecular replacement using Phaser (9), with PDB entry 5EEK used as a search model. Alternate cycles of model building and refinement were performed in Coot (10) and Refmac5 (11). The coordinate file for the ligand was generated in ACEDRG (12). The details of data collection and refinement statistics are summarized in Table S2. The atomic coordinates and electron density maps have been deposited to the PDB under accession code 9EU0. All structures complexes between enzyme and ligands were generated by Maestro (release 2024-2, Schrödinger, NY, USA).

REFERENCES

1. Motlová, L., Šnajdr, I., Kutil, Z., Andris, E., Ptáček, J., Novotná, A., Nováková, Z., Havlínová, B., Tueckmantel, W., Dráberová, H., Majer, P., Schutkowski, M., Kozikowski, A., Rulíšek, L., and Bařinka, C. (2023) Comprehensive Mechanistic View of the Hydrolysis of Oxadiazole-Based Inhibitors by Histone Deacetylase 6 (HDAC6). *ACS Chem. Biol.* **18**, 1594–1610

2. König, B., Watson, P. R., Reßing, N., Cragin, A. D., Schäker-Hübner, L., Christianson, D. W., and Hansen, F. K. (2023) Difluoromethyl-1,3,4-oxadiazoles Are Selective, Mechanism-Based, and Essentially Irreversible Inhibitors of Histone Deacetylase 6. *J. Med. Chem.* **66**, 13821–13837

3. Ripa, L., Sandmark, J., Hughes, G., Shamovsky, I., Gunnarsson, A., Johansson, J., Llinas, A., Collins, M., Jung, B., Novén, A., Pemberton, N., Mogemark, M., Xiong, Y., Li, Q., Tångefjord, S., Ek, M., and Åstrand, A. (2023) Selective and Bioavailable HDAC6 2-(Difluoromethyl)-1,3,4-oxadiazole Substrate Inhibitors and Modeling of Their Bioactivation Mechanism. *J. Med. Chem.* **66**, 14188–14207

4. Vergani, B., Sandrone, G., Marchini, M., Ripamonti, C., Cellupica, E., Galbiati, E., Caprini, G., Pavich, G., Porro, G., Rocchio, I., Lattanzio, M., Pezzuto, M., Skorupska, M., Cordella, P., Pagani, P., Pozzi, P., Pomarico, R., Modena, D., Leoni, F., Perego, R., Fossati, G., Steinkühler, C., and Stevenazzi, A. (2019) Novel Benzohydroxamate-Based Potent and Selective Histone Deacetylase 6 (HDAC6) Inhibitors Bearing a Pentaheterocyclic Scaffold: Design, Synthesis, and Biological Evaluation. *J. Med. Chem.* **62**, 10711–10739

5. Cellupica, E., Caprini, G., Cordella, P., Cukier, C., Fossati, G., Marchini, M., Rocchio, I., Sandrone, G., Vanoni, M. A., Vergani, B., Źrubek, K., Stevenazzi, A., and Steinkühler, C. (2023) Difluoromethyl-1,3,4-oxadiazoles are slow-binding substrate analog inhibitors of histone deacetylase 6 with unprecedented isotype selectivity. *J. Biol. Chem.* **299**, 1–7

6. Cellupica, E., Caprini, G., Fossati, G., Mirdita, D., Cordella, P., Marchini, M., Rocchio, I., Sandrone, G., Stevenazzi, A., Vergani, B., Steinkühler, C., and Vanoni, M. A. (2023) The Importance of the "Time Factor" for the Evaluation of Inhibition Mechanisms: The Case of Selected HDAC6 Inhibitors. *Biology (Basel).* 10.3390/biology12081049

7. Copeland, R. A. (2013) *Evaluation of Enzyme Inhibitors in Drug Discovery*, Wiley, 10.1002/9781118540398

8. Kabsch, W. (2010) XDS. *Acta Crystallogr. Sect. D Biol. Crystallogr.* **66**, 125–132

9. McCoy, A. J., Grosse-Kunstleve, R. W., Adams, P. D., Winn, M. D., Storoni, L. C., and Read, R. J. (2007) Phaser crystallographic software. *J. Appl. Crystallogr.* **40**, 658–674

10. Emsley, P., Lohkamp, B., Scott, W. G., and Cowtan, K. (2010) Features and development of Coot. *Acta Crystallogr. Sect. D Biol. Crystallogr.* **66**, 486–501

11. Murshudov, G. N., Skubák, P., Lebedev, A. A., Pannu, N. S., Steiner, R. A., Nicholls, R. A., Winn, M. D., Long, F., and Vagin, A. A. (2011) REFMAC 5 for the refinement of macromolecular crystal structures. *Acta Crystallogr. Sect. D Biol. Crystallogr.* **67**, 355–367

12. Long, F., Nicholls, R. A., Emsley, P., Gražulis, S., Merkys, A., Vaitkus, A., and Murshudov, G. N. (2017) AceDRG : a stereochemical description generator for ligands. *Acta Crystallogr. Sect. D Struct. Biol.* **73**, 112–122
